# Low-Dosage Bevacizumab Treatment: Effect on Radiation Necrosis After Gamma Knife Radiosurgery for Brain Metastases

**DOI:** 10.3389/fsurg.2021.720506

**Published:** 2021-09-03

**Authors:** Yuxiang Weng, Jie Shen, Luyuan Zhang, Zebin Fang, Feng Xiao, Chao Zhang, Zuoxu Fan, Kaiyuan Huang, Liyun Wang, Bin Huang, Fan Wu, Tiesong Zhang, Qingsheng Xu

**Affiliations:** ^1^Department of Neurosurgery, College of Medicine, The First Affiliated Hospital, Zhejiang University, Hangzhou, China; ^2^Department of Neurosurgery, Shengzhou People's Hospital, Shaoxing, China; ^3^Department of Neurosurgery, Xinchang Hospital of Traditional Chinese Medicine, Shaoxing, China

**Keywords:** low-dosage, bevacizumab, radiation necrosis, gamma knife, brain metastase

## Abstract

Cerebral radiation necrosis (RN), a complication of Gamma Knife radiosurgery, is difficult to treat, although bevacizumab seems to be effective. However, clinical data pertaining to bevacizumab treatment for RN are scarce, and its high price is problematic. This study explored the effectiveness of low-dose bevacizumab for RN caused by Gamma Knife. We retrospectively analyzed 22 patients who suffered cerebral RN post-Gamma Knife, and received bevacizumab treatment because of the poor efficacy of glucocorticoids. Low-dose bevacizumab (3 mg/kg) was administered for two cycles at 2-week intervals. T1- and T2-enhanced magnetic resonance imaging (MRI) images were examined for changes in RN status. We also monitored the dose of glucocorticoid, Karnofsky Performance Status (KPS) score, and adverse drug reactions. The mean volume of RN lesions decreased by 45% on T1-weighted images with contrast enhancement, and by 74% on T2-weighted images. All patients discontinued the use of glucocorticoids. According to the KPS scores, all patients showed an improvement in their symptoms and neurological function. No side effects were observed. Low-dosage bevacizumab at a dose of 3 mg/kg every 2 weeks is effective for treating cerebral RN after Gamma knife for brain metastases.

## Introduction

Gamma Knife, a type of stereotactic radiosurgery (SRS), is effective for local control of brain metastases (1). Unfortunately, Gamma Knife leads to the intractable complication of radiation necrosis (RN) (2). At present, glucocorticoids are the standard treatment for cerebral RN, in spite of their adverse side effects and limited treatment efficacy (3). The efficacy of other non-invasive treatments, including antiplatelet, anticoagulation and hyperbaric oxygenation, is considered controversial (1, 2, 4, 5). Surgical management can relieve clinical symptoms due to removal of the mass and reduced intracranial hypertension; however, surgery carries risks and causes neurological damage (6).

Recently, because of its ability to block vascular endothelial growth factor (VEGF), bevacizumab has proved to be an effective treatment for RN, in large dosage of 5–10 mg/kg every 2 weeks for 2–6 cycles (7–14). However, data on bevacizumab as a therapy for RN are still limited. In addition, bevacizumab is expensive, which creates a large economic burden for patients and society overall. In this study, we evaluated the efficacy of low-dosage bevacizumab treatment for RN following Gamma Knife in patients with brain metastases.

## Materials and Methods

### Patients' Characteristics

We analyzed 22 patients treated with bevacizumab for cerebral RN, caused by Gamma Knife in our center, between January 2013 and December 2017 (Table 1). All patients had metastatic brain tumors 13 from lung adenocarcinoma, 2 from Small cell lung cancer, 1 from neuroendocrine lung cancer, 2 from breast cancer, 1 from renal clear cell carcinoma, 1 from maxillary sinus carcinoma and 1 from esophageal Cancer, 1 from ovarian cancer, and long-term glucocorticoid treatment yielded unsatisfactory results.

**Table 1:** Characteristics of patients with radiation necrosis after Gamma Knife for brain metastases.

**No**.	**Sex**	**Original tumor**	**Metastatic site**	**Prescription dose (Gy)**	**% Decrease in gadolinium**	**% Decrease in T2**	**KPS change**
1	F	Lung adenocarcinoma	Left temporal and parietal	22	21	86	50
2	F	Lung adenocarcinoma	Left occipital	20	18	83	50
3	F	Lung adenocarcinoma	Right parietal	18	27	45	40
4	M	Lung adenocarcinoma	Left frontal	15	82	59	50
5	F	Lung adenocarcinoma	Right frontal and parietal	20	34	52	20
6	F	Lung adenocarcinoma	Left occipital	20	55	72	10
7	M	Lung adenocarcinoma	Right frontal	18	10	64	10
8	M	Neuroendocrine lung cancer	Right parietal	24	34	61	40
9	M	Small cell lung cancer	Left posteior horn of lateral ventricle	24	82	80	40
10	M	Small cell lung cancer	Right parietal and occipital	25	49	79	40
11	F	Breast cancer	Left frontal	24	38	81	40
12	M	Renal clear cell carcinoma	Left frontal	22	31	69	40
13	M	Maxillary sinus carcinoma	Right temporal	15	42	79	50
14	M	Esophageal cancer	Right occiptal	24	56	83	10
15	M	Lung adenocarcinoma	Right parietal	20	23	82	40
16	M	Lung adenocarcinoma	Left parietal	18	70	90	30
17	F	Lung adenocarcinoma	Right frontal	22	73	85	40
18	M	Lung adenocarcinoma	Right posteior horn of lateral ventricle	15	33	85	20
19	F	Lung adenocarcinoma	Left frontal	22	67	66	20
20	F	Lung adenocarcinoma	Right posteior horn of lateral ventricle	22	81	85	20
21	F	Breast cancer	Right frontal	15	30	78	30
22	F	Ovarian cancer	Left temporal	20	28	68	20

### Diagnostic Criteria of RN

**Figure 1:**
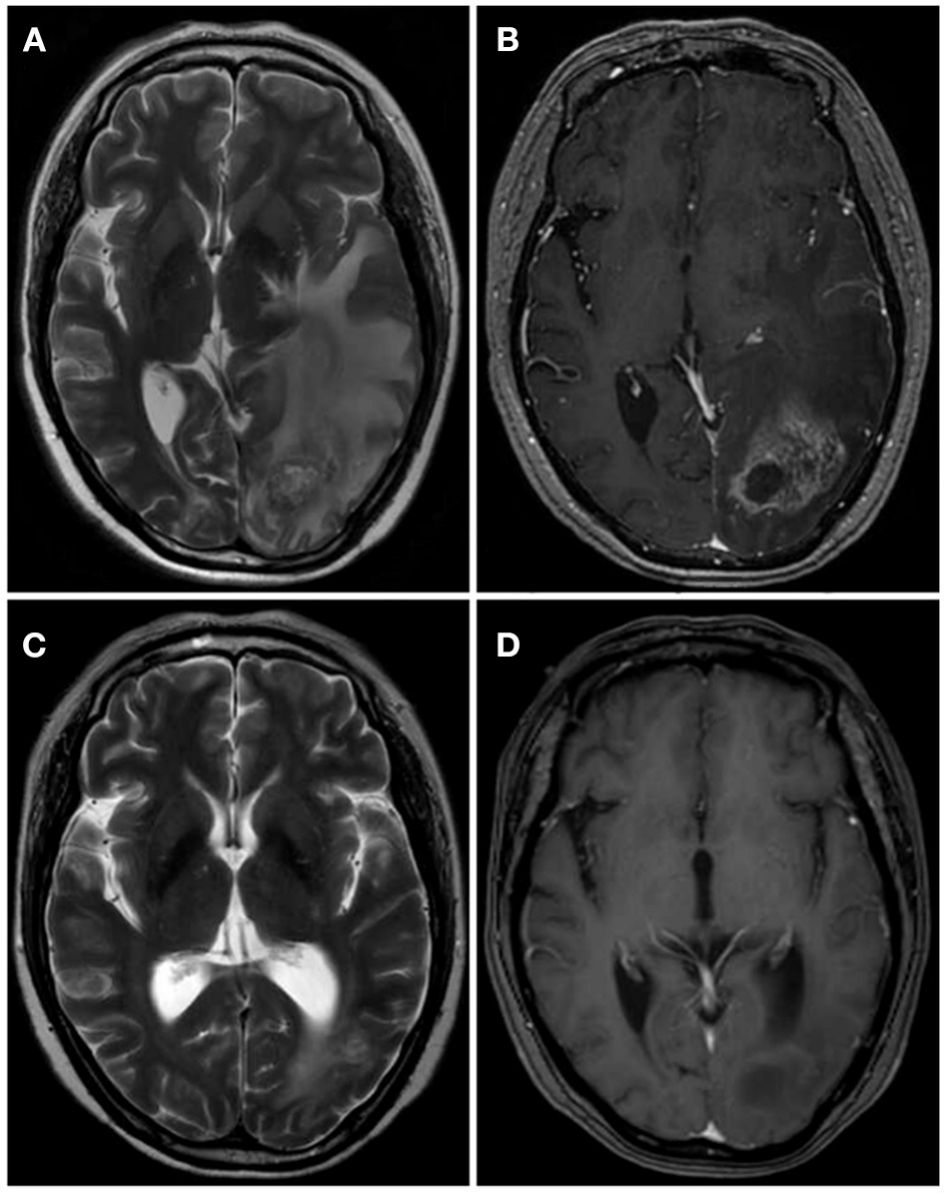
One case of RN after Gamma Knife before and after bevacizumab treatment. **(A,B)** MRI showed the lesion was located in left occipital with large edema area and obvious enhancement surrounding the tumor. **(C,D)** After the therapy of bevacizumab (3 mg/kg) for 2 cycles, the zone of lesion reduced dramatically.

Pathology is the gold standard for a diagnosis of RN, although it is difficult to obtain biopsy specimens. Therefore, RN is usually diagnosed with the help of medical imaging (14, 15). In our study, the final diagnosis of RN was based on history of receiving Gamma Knife, clinical symptoms, physical examination, imaging, and biopsy. Low T1-weighted and high T2-weighted magnetic resonance imaging (MRI) images revealed cerebral edema surrounding the tumor and an enhanced irradiated field (Figure 1). Perfusion computed tomography (PCT) demonstrated low cerebral blood volume (CBV), low cerebral blood flow (CBF) and higher mean transit time (MTT) in RN (16).

### Therapeutic Regimen Based on Bevacizumab

All patients received two low dosage of bevacizumab (3 mg/kg), 2 weeks apart, for 2–4 courses. Two patients had two separate cycles of bevacizumab because RN recurred during the 6 months follow-up after bevacizumab discontinuation. All patients were hospitalized for observation of adverse drug reactions.

### Treatment Assessment

Enhanced MRI was performed before and after bevacizumab treatment, and was then repeated 2 months after the end of bevacizumab therapy in the two patients with repeated cycles. When patients developed neurological symptoms, MRI scans were evaluated immediately. We calculated the volume of RN lesions on T1 and T2 sequences, and compared the findings to those based on images acquired before bevacizumab was started. When patients underwent MRI scans pre- and post-bevacizumab, or to check for the onset of new symptoms, we could analyze the clinical data in terms of changes in neurological symptoms, glucocorticoid dosage, Karnofsky Performance Status (KPS) score, and clinical outcome.

## Results

**Figure 2:**
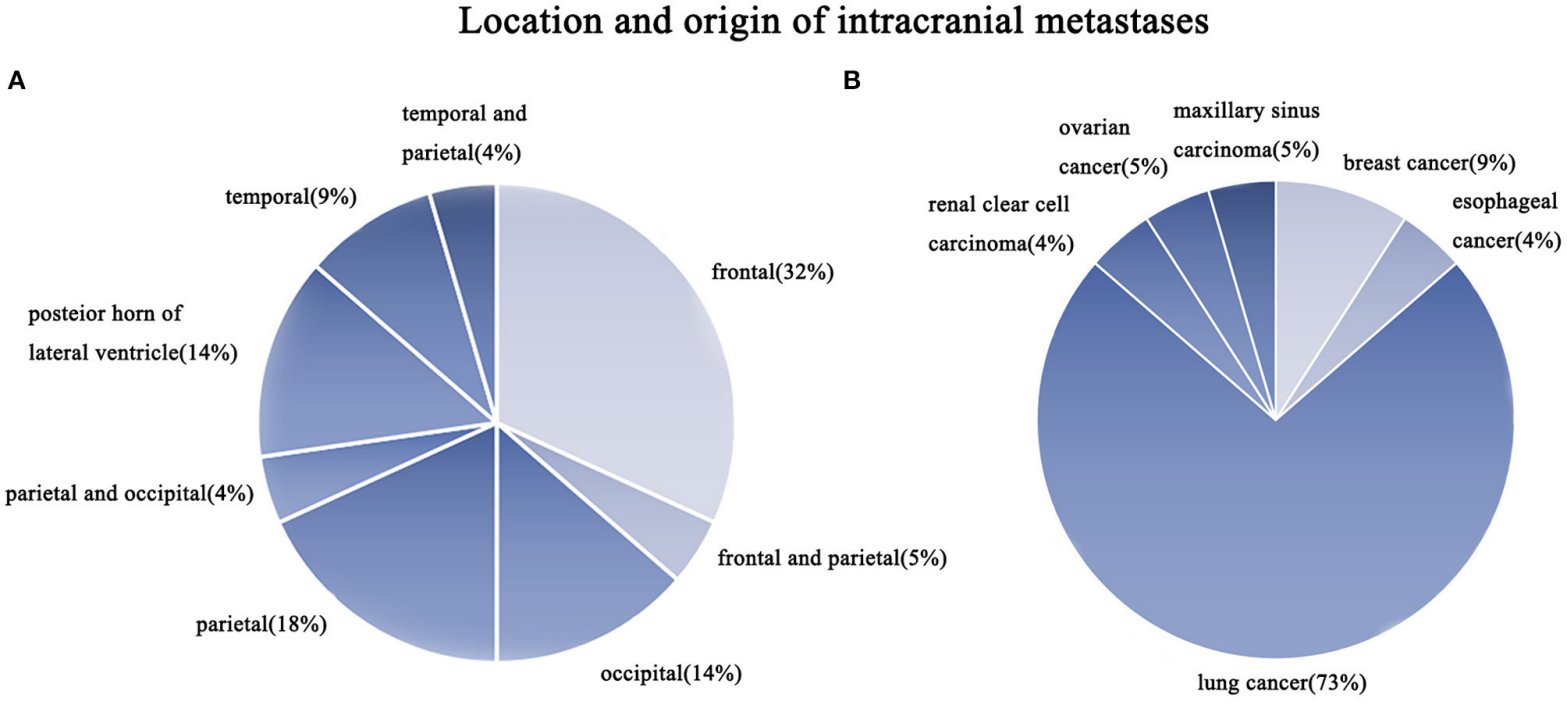
**(A)** The location of the intracranial metastases in this series of cases, with the frontal lobe having the most lesions, followed by the temporal lobe. **(B)** The source of metastases in this series of cases, with lung cancer having the most brain metastases (73%).

**Figure 3:**
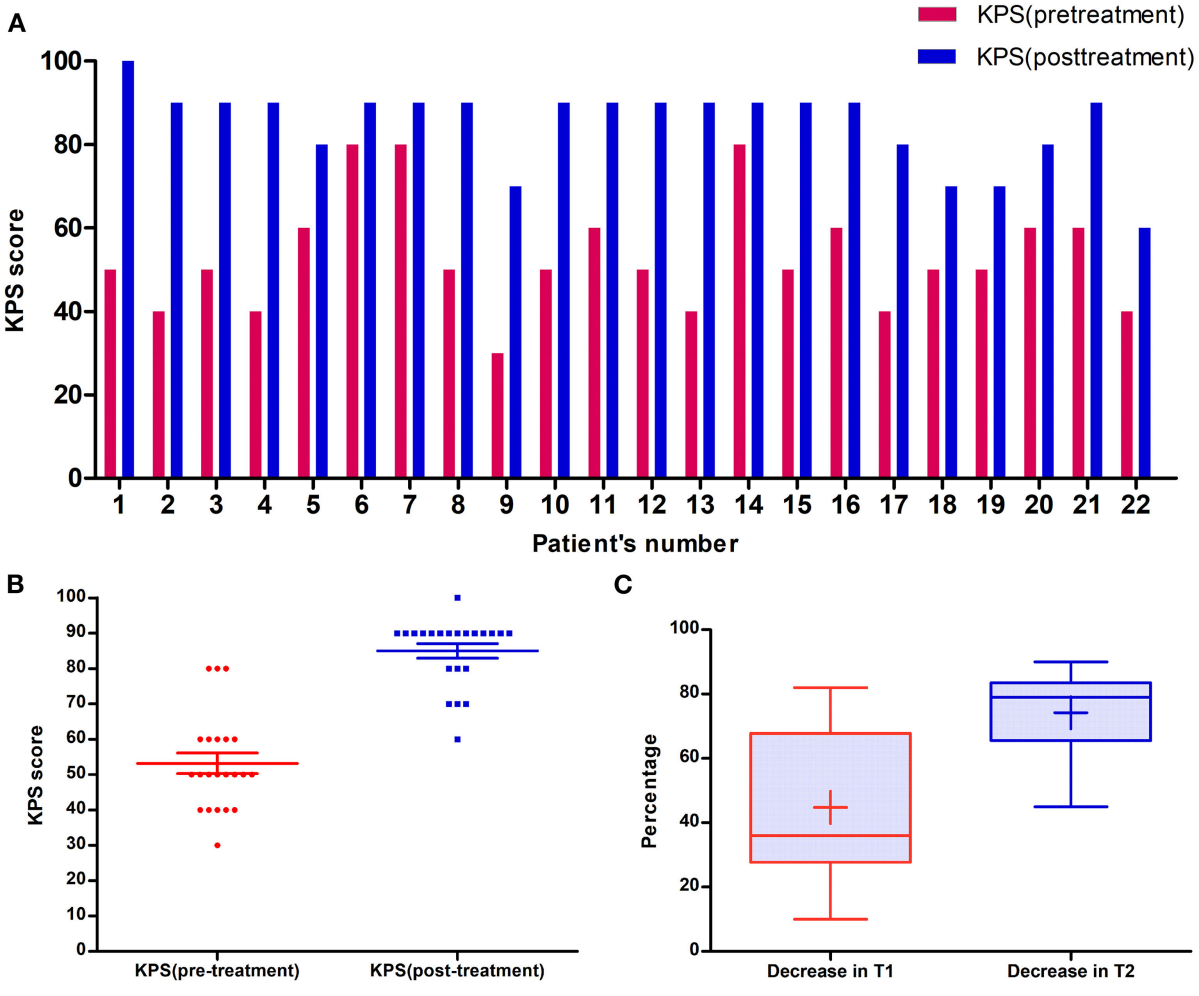
**(A)** The change of each patient's KPS score after bevacizumab treatment. **(B)** Scatter plot of KPS scores of patients in cohort before and after bevacizumab treatment: the median KPS score of patients before bevacizumab treatment was 50, however, median KPS score increased to 90 after bevacizumab treatment. **(C)** Boxplot of imageology of patients in cohort before and after bevacizumab treatment. After bevacizumab treatment, the volume of nidus in Gadolinium and T2 sequence decreased by 44.7 and 74.2%, respectively.

Table 1 shows patients ranged in age from 48 to 79 years (median: 64 years old). Eleven patients were male and others were female; all patients had undergone Gamma Knife with the prescription dosage of 15–25 Gy (median dosage: 20 Gy). In these patients, sixteen (72.7%) patients had brain metastases from lung cancer (Figure 2). Among 22 patients, 7 (31.8%) patients had metastases located in the frontal lobe, and 4 (18.2%) patients had metastases located in the parietal lobe (Figure 2). The mean volume of the RN lesions was 14.5 cm^3^ (range from 3.1 to 45.9 cm^3^) on T1 images post-gadolinium, and 109.25 cm^3^ (range: 26.7–266.8 cm^3^) on T2 images before bevacizumab treatment. After bevacizumab therapy, the average lesion volume was 6.99 cm^3^ (range: 0.7–20.6 cm^3^) on T1 contrast images and 26.1 cm^3^ (range: 5.6–60.8 cm^3^) on T2 images. Therefore, the average volume of RN lesions was reduced by 45% on T1-weighted contrast images and 74% on T2-weighted images (Figure 3). All patients discontinued the use of glucocorticoids after receiving two cycles of bevacizumab therapy. KPS scores were increased by an average of 31.8 in all patients' post-therapy (Figure 3). No side effects were observed.

## Discussion

RN is the most common complication in patients with brain metastases treated by SRS (2). The main factors associated with RN are irradiation dosage, treatment duration, volume of irradiation and fractionation regimen (17, 18). RN usually occurs 3 or more months after radiotherapy (mean: 11.6 months) and 13–17% of patients develop some degree of RN after receiving SRS therapy for 1 year (19, 20). A meta-analysis of RN found that the most common necrotic sites were the frontal lobe (25%), temporal lobe (24%), and parietal lobe (10%). RN causes symptoms including focal or systemic neurological deficits. In severe cases, patients may experience a major decrease in quality of life (17, 21).

The mechanism of RN is unclear. At present, it is believed that RN progresses continuously from endothelial cell dysfunction to tissue hypoxia and necrosis, accompanied by the release of cytokines, such as VEGF (2, 14, 17, 18), which can lead to destruction of the blood-brain barrier permeability and cerebral edema (22, 23). Both animal and human models of RN have shown that high levels of VEGF occur due to blood-brain barrier dysfunction (24, 25). Nordal et al. reported that lab rats without the gene encoding VEGF were more resistant to radiation damage (26). Early blocking of VEGF can lower the risk of RN by reducing vascular permeability. This may help reverse pathological mechanisms, improve symptoms and prevent further disease progression. Bevacizumab seems to be an effective new treatment for RN, which exerts its effects through angiogenesis inhibition.

Data on the efficacy of bevacizumab treatment for RN are limited. However, recent literature has suggested that bevacizumab plays an important role in preventing RN. Gonzalez et al. were the first to report a potential benefit of bevacizumab treatment on RN. After treatment with bevacizumab at a dosage of 5 mg/kg every 2 weeks, or 7.5 mg/kg every 3 weeks, the neurological symptoms of eight patients affected by cerebral RN improved. The average reduction in the abnormal area on T1-weighted post-gadolinium images was 48%, compared to 60% on fluid-attenuated inversion recovery (FLAIR) images (3). Torcuator et al. assessed six biopsy-confirmed RN patients who received low-dosage bevacizumab; follow-up MRI showed an improvement in RN, with an average reduction in the abnormal area on T1-weighted post-gadolinium and FLAIR images of 79 and 49%, respectively (27). In a randomized, double-blind, placebo-controlled study, after bevacizumab treatment (7.5 mg/kg every 3 weeks) 14 patients with biopsy-confirmed RN showed a median reduction in edema volume of 59 and 63% on T2-FLAIR and T1-weighted post-gadolinium images, respectively. In addition, although some complications occurred after bevacizumab treatment, these 14 patients showed improvements in clinical symptoms (15). Delishaj et al. reviewed 125 patients, 114 (91.2%) of whom showed an improvement in neurological symptoms (17). In our study, the mean lesion volume decreased by 41 and 71% on T1-weighted contrast images and T2-weighted images, respectively. The KPS scores of all patients improved by > 30. Previous studies reported that patient dependence on glucocorticoids decreased after bevacizumab treatment, and that the dosage of glucocorticoids could be reduced (7, 23, 28). In our center, all patients gradually tapered off glucocorticoids after two cycles of bevacizumab treatment. Our data are consistent with previous studies, and therefore support a role of bevacizumab in the treatment of RN after Gamma Knife treatment for cerebral metastases.

In a prospective phase II clinical study, bevacizumab at a dosage of 1 mg/kg was used to treat cerebral RN. The regimen included three treatment cycles and one infusion every 3 weeks. Preliminary results showed that the severity of the symptoms decreased after bevacizumab treatment in 90% of patients (29). This suggests that ultra-low dosage bevacizumab could be a valid alternative to the standard dosage. At present, bevacizumab is regarded as a treatment that can be applied subsequent to RN diagnosis; whether it could also be used as a preventive treatment is rarely mentioned. A study including 54 adults male Wistar rats reported less severe RN in animals receiving prophylactic bevacizumab treatment before a 100-Gy radiation dosage (30). If further clinical research confirms these findings, it may be possible to consider bevacizumab as a preventive rather than purely treatment modality.

Bevacizumab has few side effects; however, patients who receive this agent are at risk of gastrointestinal perforation, delayed wound healing, thromboembolic events, asymptomatic ischemic changes, and even symptomatic ischemic changes such as hemiplegia (5, 31). The rate of adverse events (9.5%) reported by Bodensohn et al. (23) was not replicated in our study; in fact, no adverse events were observed in any of the 14 patients treated with low-doage bevacizumab. However, this finding is likely related to the small number of patients and relatively short follow-up time.

Our study had several limitations, including the small sample, which was recruited from a single-center with a descriptive analysis. This may have biased the analysis and conclusions. Larger studies are needed to confirm our findings and determine the optimal bevacizumab treatment schedule for RN.

## Conclusions

In conclusion, low-dosage bevacizumab at a dosage of 3 mg/kg every 2 weeks is effective for the treatment of cerebral RN after Gamma Knife for brain metastases.

## Data Availability Statement

The original contributions presented in the study are included in the article/supplementary material, further inquiries can be directed to the corresponding author/s.

## Ethics Statement

Ethics approval has been obtained from the ethics committee of the First Affiliated Hospital of Zhejiang University. The patients/participants provided their written informed consent to participate in this study.

## Author Contributions

QX is guarantor of integrity of the entire study. YW, JS, and LZ designed the study and prepare manuscript. ZFang and CZ collected the patient's information. FX, ZFan, and KH are response to statistical analysis. BH, LW, and TZ contributed to literature research. All authors agreed to be accountable for the content of the work.

## Conflict of Interest

The authors declare that the research was conducted in the absence of any commercial or financial relationships that could be construed as a potential conflict of interest.

## Publisher's Note

All claims expressed in this article are solely those of the authors and do not necessarily represent those of their affiliated organizations, or those of the publisher, the editors and the reviewers. Any product that may be evaluated in this article, or claim that may be made by its manufacturer, is not guaranteed or endorsed by the publisher.
